# QTL Hotspots for Early Vigor and Related Traits under Dry Direct-Seeded System in Rice (*Oryza sativa* L.)

**DOI:** 10.3389/fpls.2017.00286

**Published:** 2017-03-02

**Authors:** Uma M. Singh, Shailesh Yadav, Shilpi Dixit, P. Janaki Ramayya, M. Nagamallika Devi, K. Anitha Raman, Arvind Kumar

**Affiliations:** ^1^Department of Plant Breeding, International Rice Research Institute-South Asia Hub, ICRISATPatancheru, India; ^2^Department of Plant Breeding, International Rice Research InstituteMetro Manila, Philippines

**Keywords:** direct-seeded rice, early vigor, quantitative trait loci, QTL hotspots, candidate genes, single nucleotide polymorphism

## Abstract

Strong seedling vigor is desirable trait in dry direct-seeded rice (DSR) for enhancing crop establishment and the ability to compete against weeds. A set of 253 BC_3_F_4_ lines derived from cross between Swarna and Moroberekan was phenotyped for early vigor (EV) and 8 related traits *viz*., early uniform emergence (EUE), shoot length (SHL), stem length (SL), shoot fresh weight (SFW), total fresh weight (TFW), shoot dry weight (SDW), total dry weight (TDW), and root dry weight (RDW). Composite interval mapping analysis using genotypic data from 194 SNP markers identified six genomic regions associated with traits on chromosomes 3, 4, 5, and 6 with phenotypic variance ranging from 2.5 to 18.6%. Among them 2 QTL regions; one on chr3 (id3001701-id300833) and the other on chr5 (wd5002636-id5001470) were identified as QTL hotspots A and B respectively and expressed consistently in field as well as glasshouse condition. The majority of QTLs identified for early vigor, and related traits were clustered in the QTL hotspots A (*qEV_3.1_, qEUE_3.1_, qSHL_3.1_, qSL_3.1_, qSFW_3.1_, qTFW_3.1_, qRDW_3.1_*) and QTL hotspot B (*qEV_5.1_, qEUE_5.1_, qSHL_5.1_, qSL_5.1_, qSFW_5.1_, qSDW_5.1_, qTDW_5.1_*). Ten putative candidate genes *viz*., 1-alpha-amylase precursor, 2-glutamate decarboxylase, 1-ethylene-insensitive 3, 3-expansin precursor, and 3-phenylalanine ammonia-lyase associated with the target traits were identified in the selected QTL regions. Mutations were identified in the coding region of alpha-amylase precursor and ethylene-insensitive 3 gene between the parents which can be utilized in marker assisted breeding. Trait relationships among the agro-physiological traits were examined to select the best genotypes for the given traits for use in future breeding programs.

## Introduction

Rice is the most grown cereal worldwide and is the major food for more than one-third of the world's population. Majority of the rice growing areas in Asia are occupied by transplanted-puddled rice (TPR; Pandey and Velasco, 2005) which requires the transplantation of seedlings into puddled soil. By creating an anaerobic condition, TPR has the advantages of easy seedling establishment, restrained weed growth, and enhanced nutrient availability (Singh et al., 2001). However, TPR requires large amounts of water, labor, and energy. A shortage of these inputs will make rice production through TPR more expensive, less profitable, and unsustainable (Farooq et al., 2011). Moreover, the increasing instances of the delayed arrival of monsoon as well as the decreasing amount of initial rains lead to delayed transplanting of over-aged seedlings or crop failure at nursery stage. DSR could be an alternative strategy for sustainable rice cultivation and to overcome labor, water, and energy shortages. It is a cultivation method that is very timely under the scenario of increasing global warming. Reports have suggested that DSR has the advantage of input water saving from 35 to 57%, low labor costs, and low methane emission (Sharma et al., 2002; Singh et al., 2002; Sidhu et al., 2014). Given these factors, a shift from TPR to DSR in the rice cultivation system is deemed necessary. Direct seeding can be categorized as (1) Dry DSR where dry seeds are mechanically/manually sown on dry soil, (2) Wet DSR where sprouted seeds are broadcasted on wet soil; and (3) Water DSR where seeds are broadcasted in standing water (Mahender et al., 2015). Dry DSR is more advantageous over wet and water DSR as it consumes less water, less labor intensive, saves time in sowing the crop, has low methane emission (Chauhan, 2012; Joshi et al., 2013), and is better suited to undertake mechanized agricultural operations.

Despite its advantages over TPR, however, DSR has major drawbacks such as the non-uniform emergence and uneven population of seeds in the field as well as high weed growth (Chauhan and Abugho, 2013). Seedlings with rapid uniform emergence and strong seedling vigor can access nutrients effectively and suppress the growth of weeds. Previous reports have suggested that seeds with high vigor always have more uniform emergence than those with low vigor (Egli and Rucker, 2012). The ability of a plant's aerial part to emerge rapidly from soil or water is known as early vigor (Heydecker, 1960). The strong early vigor of seedlings is crucial for their establishment and their eventual success in terms of biomass production or yield. Good early vigor as a seedling property is paramount in low temperature prone rice growing areas such as temperate or tropical and sub-tropical areas at high elevations with cold water supply (Redona and Mackill, 1996). Genotypes with early uniform emergence and strong early vigor can produce uniform plant population in the field and can suppress weed growth, thereby, providing the roots with better access to soil water and nutrients (Zhao et al., 2006; Finch-Savage et al., 2010). However, the improvement of rice for uniform emergence and early vigor *via* conventional breeding is difficult due to their quantitative inheritance (Redona and Mackill, 1996).

Rice gene pool is rich in genetic variation for early uniform emergence as well as early vigor (Redona and Mackill, 1996; Zhao et al., 2006; Namuco et al., 2009) and this variation could be exploited in identifying and introgressing favorable alleles for selected traits through marker-assisted breeding. Uniform emergence and early vigor are complex traits which are influenced by different factors such as seed vigor (ability of the seed to grow), heterotrophic (seed reserve) and autotrophic growth (photosynthesis), and environmental conditions, among others. Many efforts have been made to improve these traits by providing good agronomic conditions but the approach has now shifted toward investigating the genetic basis and using it in marker-assisted breeding programs. Studies have also been undertaken to identify quantitative trait loci (QTLs) for seed emergence and early vigor traits in wheat (Maydup et al., 2012; Bai et al., 2013; Moore and Rebetzke, 2015), Arabidopsis (Galpaz and Reymond, 2010; DeRose-Wilson and Gaut, 2011), tomato (Foolad et al., 2007; Khan et al., 2012), soy bean (Csanádi et al., 2001), barley (Mano and Takeda, 1997), and *Brassica napus* (Hatzig et al., 2015). These studies have reported that early vigor traits of seedlings are regulated by a cluster of genes and are strongly affected by environmental conditions (Bettey et al., 2000; Koornneef et al., 2002; Finch-Savage et al., 2010).

Identification of QTLs associated with early vigor in rice has been undertaken using different types of mapping population (Lu et al., 2007; Sandhu et al., 2014). QTLs for related traits such as shoot length, stem length, shoot fresh weight (SFW), shoot dry weight (SDW), and root dry weight (RDW) have also been mapped on rice chromosomes (Redona and Mackill, 1996; Cui et al., 2002, 2008; Xu et al., 2004; Zhang et al., 2005a,b; Kanbar et al., 2006; Zhou et al., 2007; Cairns et al., 2009; Xie et al., 2014). However, only one study (Cairns et al., 2009) was reported on combined QTL analysis for early vigor and related traits in both field as well as controlled condition. The combined analysis will help in detecting QTLs that consistently express across environments as well as the key component traits that are important for dry DSR cultivation system at the early seedling stage. Therefore, this study aims to (1) detect QTLs responsible for early vigor and related traits; and (2) study the correlations between physiological and agronomical traits to select best genotypes for dry direct-seeded rice.

## Materials and methods

The experiment was carried out at the International Rice Research Institute—South Asia Hub (IRRI-SAH), Patancheru (78° 16′ longitude, 17° 32′ latitude and 540 m above sea level) during wet season (WS) 2014 under field condition and during WS2015 under field and glasshouse conditions. Experimental site was typically semi-arid tropic in nature with alfisols soil type (Supplementary Table 1).

### Plant materials

The mapping population, Swarna^*^3/Moroberekan (Dixit et al., 2014) used in the study included a set of 342 BC_3_F_4_ derived-backcross lines (BLs) developed by single seed descent (SSD) method from a cross between Swarna as recipient (lowland indica rice variety) and Moroberekan as donor (upland traditional japonica rice variety). Moroberekan possesses long shoots and a deep root system and is a sturdy plant type. On the other hand, Swarna possesses fast seedling germination and high tiller number. Prior to the experiment, the seed germination of a random sample of 10 entries was examined under 32°C and a germination rate of 100% at 5 days after incubation was observed. Dry seed was sown by dibbling seed manually in furrow and the furrow was closed by running a blade harrow. However, when planted in the field, 89 of the 342 entries showed poor germination and were excluded from the analysis. The seeds for the WS2015 field and glasshouse experiments were obtained from the WS2014 field experiment.

### Field experiment

Mapping population was evaluated during WS2014 and WS2015 for early vigor, and agronomic traits under DSR conditions using an alpha lattice design in two replications at spacing of 20 × 15 cm with plot size of 4 m^2^. Field was irrigated based on soil moisture levels recorded by tensiometers (12–14 Kpa) fixed at various points in the experimental field. Pre-emergence herbicide pendimethalin (Stomp) @ 2 ml/L was sprayed on the next day of sowing followed by the post-emergence herbicide bispyribac-sodium (Nominee Gold) @ 2 ml/L after 21 DAS and then followed by two manual weeding during the cropping period. Fertilizers were applied at a rate of 100:60:40 kg NPK/ha. Full doses of phosphorus (P) and potassium (K) were applied during sowing while N was divided into three equal splits i.e., one-third applied at the time of sowing, one-third just after first weeding (25–30 days of crop age), and one-third at the panicle initiation stage. Early vigor was evaluated twice in WS2014 (June and August 2014) and once in WS2015 (June 2015) at 30 DAS following the Standard Evaluation System (SES) of IRRI (1996)—1, for a very fast growing plant (5–6 leaves); 3, fast growing plant (4–5 leaves); 5, normal plant (4 leaves); 7, weak plant (3–4 leaves); and 9, very weak plant (yellow leaf). Plant height (cm) was measured from randomly selected 3 plants and grain yield (kg ha^−1^) are measured from 2 rows (4 m^2^ each) from each replication.

### Glasshouse experiment

The glasshouse experiment was conducted during WS2015. Day/night temperature (30/25°C), relative humidity (80–90%) and day/night length (13/11 h) remained same for entire duration of the experiments. Early uniform emergence study was carried out using the germination test technique with the seeds grown in a petri plate (22.5 cm width) containing the wet foam paper in the replicated trial. EUE was recorded when all plumules became visible and their lengths were approximately equal to the seed length. The experiment for the remaining early vigor related traits like shoot length, stem length, total fresh weight (TFW), SFW, total dry weight (TDW), SDW, and RDW was conducted in trays, filled with sand: clay at the ratio of 4:1, without any fertilizer application. Each tray contained 13 rows and 8 columns. Seeds were sown at 1 cm soil depth. Trays were watered every day to ensure an adequate supply of water. Replicates were sown in the trays on 1 day interval. The measurement of SHL, SL, TFW, SFW, TDW, SDW, and RDW were taken from three plants of each replicates at 8 and 21 DAS. Dry weights were observed for shoot, root, and total plant by oven drying the sample at 80°C for 72 h_._

### Genotyping, construction of linkage map, and QTL mapping

SNP marker analysis was performed and a linkage map was constructed (Dixit et al., 2014; Supplementary Table 4), which consisted of 194 marker loci covering all 12 chromosomes and spanning 1,525 cM with an average interval of 7.86 cM between markers. QTL analysis was done using QGene 4.3.10 with the composite interval mapping procedure (Joehanes and Nelson, 2008). Standard threshold LOD (logarithm of odds) score of 2.5 was used to suggest the presence of putative QTL (Ramayya et al., 2016). The candidate genes for early vigor, and related traits were identified based on available literature and on the Gramene database.

### Identification of putative candidate genes and mutations

Sequences of SNPs flanking QTLs for early vigor and related traits on chromosome 3, 4, 5, and 6 were subjected to BlastN on the RicePlex database. The outputs were used to retrieve putative candidate genes. These genes have been analyzed between parents to identify SNPs by using OryzaSNP browser.

### Agro-physiological data analysis

Agronomic data from the field experiment of WS2014 and WS2015 were analyzed using a mixed model, taking lines as fixed and replicates and blocks within replicates as random. The means were estimated using the MIXED procedure of SAS (Littell et al., 2006). A variance component analysis is also performed for each trial using the above model except that the genotypes are set to random in order to estimate the repeatability (broad-sense heritability, hence forth referred to as *H*). *H* is calculated using the following formula

$$\begin{array}{l} H=\frac{\sigma_{g}^{2}}{\sigma_{p}^{2}}\text{ where }\sigma_{p}^{2}=\sigma_{g}^{2}+\left(\frac{\sigma_{e}^{2}}{r}\right) \end{array}$$

$\sigma_{g}^{2}$ is the genotypic variance, $\sigma_{p}^{2}$ is the phenotypic variance, $\sigma_{e}^{2}$ is the error variance and *r* the number of replicates. Multi-dimensional preference analysis (MDPREF) was then conducted (Caroll, 1972) to explore the agronomic and physiological traits that correlated with early uniform emergence and early vigor as well as the pattern of genotype differences for the given traits. MDPREF is a principal component analysis (PCA) of the qualitative data performed using the PRINQUAL procedure of SAS (SAS Institute, 1996; Linting et al., 2007). The PRINQUAL procedure transforms the qualitative traits to maximize the fit of the data to the linear principal components model. In the current case, a monotonic transformation was applied to the data on early vigor owing to its ordinal nature (Kruskal and Shepard, 1974). A MDPREF biplot with genotypes in rows and traits in columns best represents a summary of the relationships among the genotype scores on a two-dimensional space while at the same time plotting into this a configuration vector for each trait. MDPREF identifies the variability that is most salient to the preference patterns of the traits toward the genotypes and extracts this as the first principal component. At one end of the plot of the first principal component are the most preferred genotypes for the respective traits; at the other end are the least preferred genotypes with respect to the first component. The second principal component represents the direction that is most salient to the preferences that are orthogonal to the first principal component (Kuhfeld, 1992).

## Results

### Analysis of variance of agronomic and physiological traits

Mixed model analysis of data from the field trials conducted during the wet seasons of 2014 and 2015 revealed significant differences between the entries for plant height and grain yield (kg ha^−1^). Analysis of variance of the physiological traits measured on 8 days after sowing (DAS) and 21 DAS for the glasshouse trial also indicated that the entries differed significantly for most traits except SDW on both measurement days and RDW at 21 DAS (Supplementary Table 2). The mean of agronomic traits of the genotypes and statistical differences between the traits tested in 2014 and 2015 is analyzed. The heritability for days to flowering was 0.88 and 0.90 (2014 and 2015); plant height was 0.84 and 0.69 (2014 and 2015); and that of grain yield was 0.66 and 0.69 (2014 and 2015; Supplementary Table 3).

### QTL analysis

#### Identification of QTLs for early vigor and related traits

**Figure 1:**
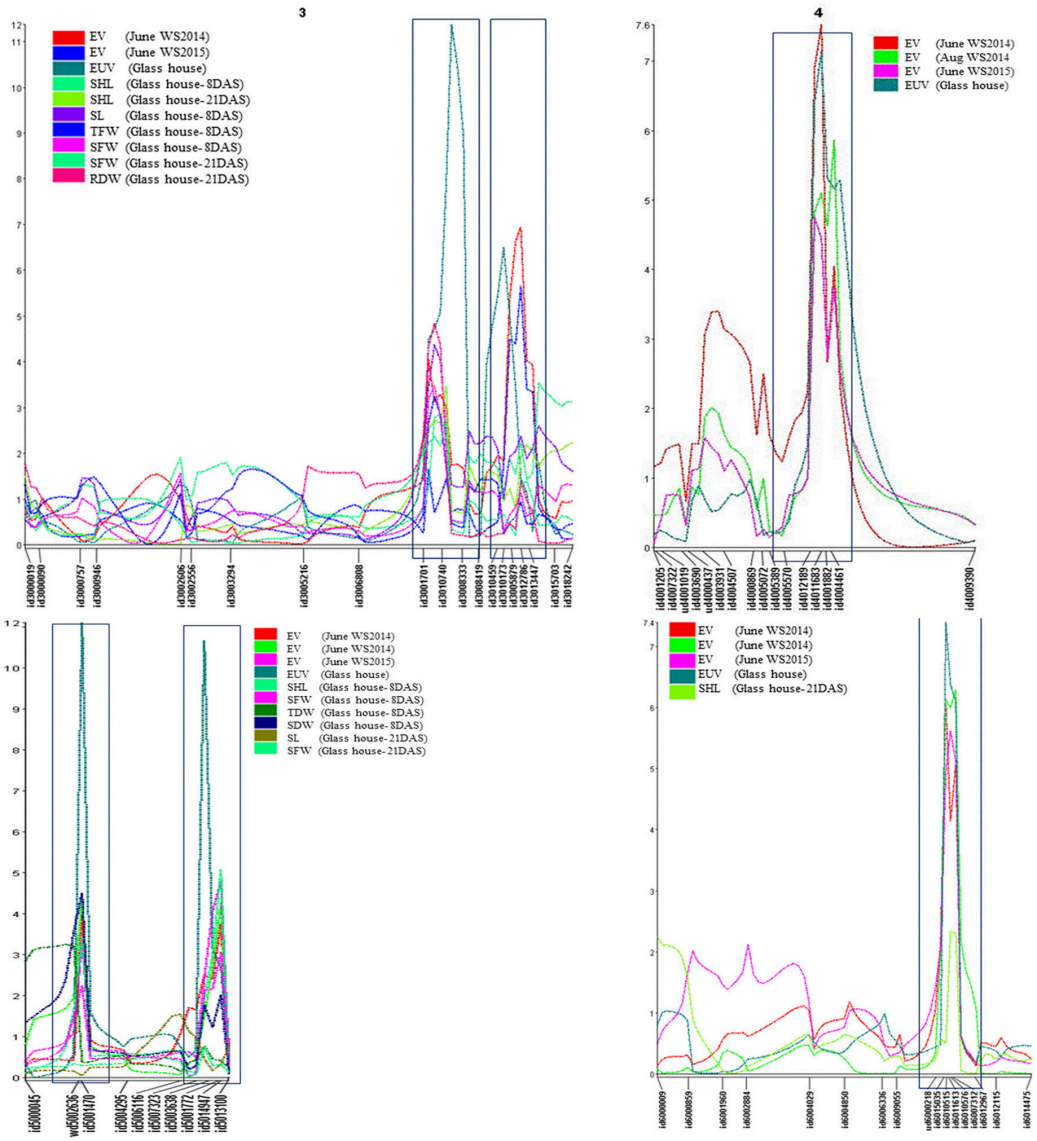
**Co-localization of QTLs for early vigor, early uniform emergence and related traits on chromosome 3, 4, 5, and 6**. EV, early vigor; EUE, early uniform emergence; SHL, shoot length; SL, stem length; SFW, shoot fresh weight; TFW, total fresh weight; SDW, shoot dry weight; RDR, root dry weight; TDW, total dry weight.

**Figure 2:**
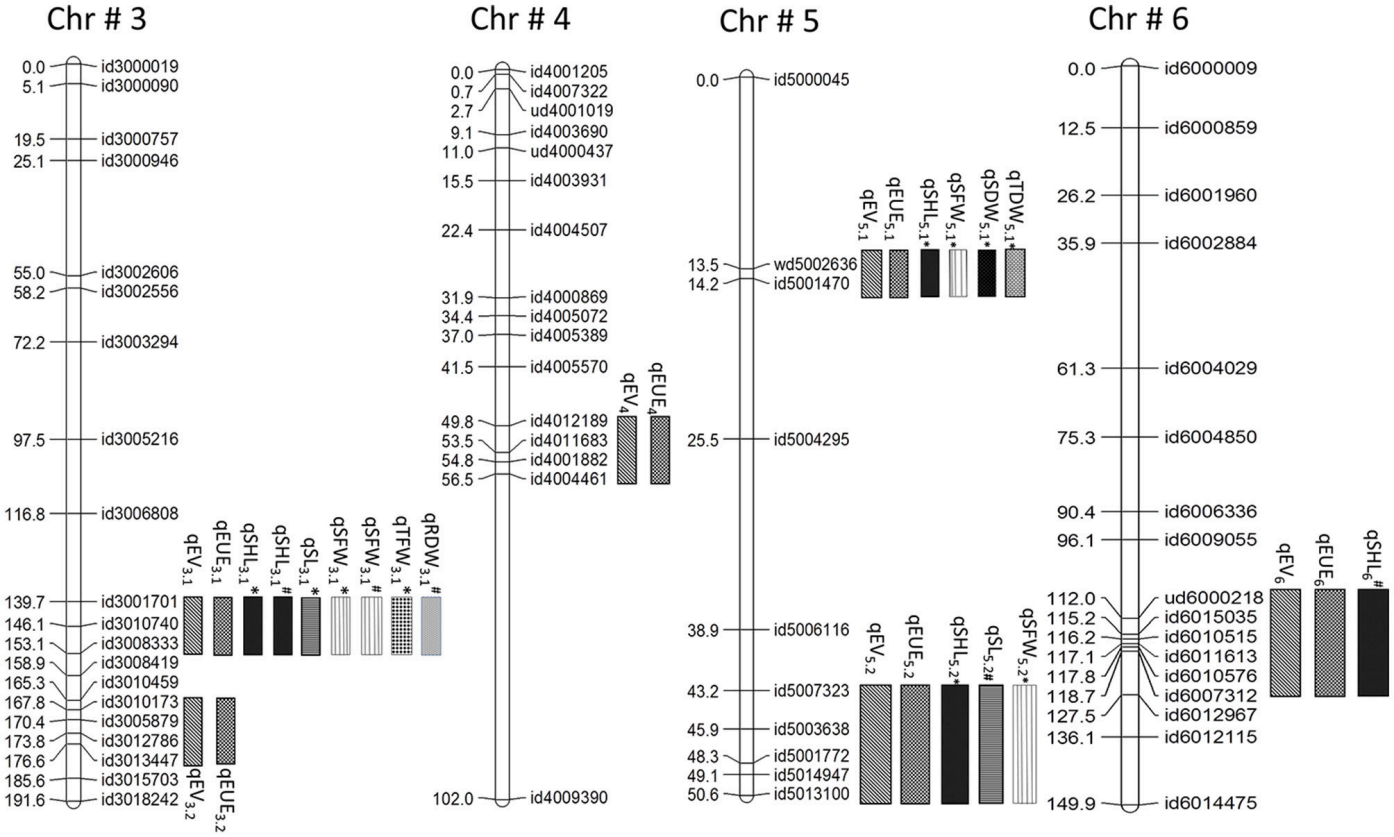
Co-localization of QTLs for early vigor (EV), early uniform emergence (EUE), shoot length (SHL), stem length (SL), shoot fresh weight (SFW), total fresh weight (TFW), shoot dry weight (SDW), total dry weight (TDW), root dry weight (RDW) in 3^*^Swama/Moroberekan population. ^*^At 8 DAS; #at 21 DAS.

QTL analysis based on early vigor measured at 30 DAS identified 17 QTLs (Supplementary Table 5). Only those QTLs that expressed in multiple environments and also in glasshouse experiments are discussed (Table 1). This includes, 2 on chromosome 3 (*qEV*_*3.1*_ and *qEV*_*3.2*_), 1 on chromosome 4 (*qEV*_*4.1*_), 2 on chromosome 5 (*qEV*_*5.1*_ and *qEV*_*5.2*_), and 1 on chromosome 6 (*qEV*_*6.1*_). These QTLs explained maximum phenotypic variances of 7.2, 13.1, 12.1, 7.8, 7.5, and 10.6%, respectively (Figures 1, 2, Table 1). QTL analysis based on the glasshouse experiment identified several QTLs for 8 early vigor related traits (Supplementary Table 5). However, only those QTLs that coincide with QTL of field experiment are discussed (Table 1). The QTLs for early uniform emergence were detected on chromosome 3 (*qEUE*_*3.1*_ and *qEUE*_*3.2*_), chromosome 4 (*qEUE*_*4.1*_), chromosome 5 (*qEUE*_*5.1*_ and*qEUE*_*5.2*_), and chromosome 6 (*qEUE*_*6.1*_), which explained phenotypic variances of 18.6, 11.2, 12.2, 18.3, 12.6, and 12.5%, respectively.

**Table 1:** QTL analysis for early vigor and related traits in 3^*^Swarna/Moroberekan population of rice.

**Chr^a^**	**Nearest marker**	**Marker interval**	**Position (cM)^b^**	**QTL**	**Traits/Environment**	**Peak LOD^c^**	***R*****^2^^d^**	**Additive effect^e^**	**Donor of positive allele**
3	id3010740	id3001701-id3008333	3.2–24.5	*qEV_*3.1*_*	EV1	4.13	7.2	0.91	SW
				*qEV_*3.1*_*	EV3	3.2	5.8	1.08	SW
				*qEUE_*3.1*_*	EUE	11.3	18.6	3.715	SW
				*qSHL_*3.1*_*	SHL-8DAS	2.8	5.1	−0.113	MO
				*qSHL_*3.1*_*	SHL-21DAS	3.5	6.1	−1.44	MO
				*qSL_*3.1*_*	SL-8DAS	4	7	−0.219	MO
				*qSFW_*3.1*_*	SFW-8DAS	3.4	6.1	−0.001	MO
				*qSFW_*3.1*_*	SFW-21DAS	2.7	4.8	−0.008	MO
				*qTFW_*3.1*_*	TFW-8DAS	3.2	5.7	−0.001	MO
				*qRDW_*3.1*_*	RDW-21DAS	4.8	8.4	−0.001	MO
3	id3005879	id3010173-id3013447	11.3–29.5	*qEV_*3.2*_*	EV1	7.7	13.1	1.17	SW
				*qEV_*3.2*_*	EV2	7.43	12.6	2.039	SW
				*qEV_*3.2*_*	EV3	6.8	11.7	1.26	SW
				*qEUE_*3.2*_*	EUE	6.49	11.2	2.15	SW
4	id4011683	id4012189-id4004461	16.2–35.3	*qEV_*4.1*_*	EV1	7.6	12.1	2.04	SW
				*qEV_*4.1*_*	EV2	5.6	9.7	1.40	SW
				*qEV_*4.1*_*	EV3	4.4	7.8	1.459	SW
				*qEUE_*4.1*_*	EUE	7.1	12.2	2.464	SW
5	wd5002636	wd5002636-id5001470	2.5–19.5	*qEV_*5.1*_*	EV1	4.2	7.4	1.95	SW
				*qEV_*5.1*_*	EV2	4.4	7.8	2.10	SW
				*qEV_*5.1*_*	EV3	3.37	6.0	1.587	SW
				*qEUE_*5.1*_*	EUE	11	18.3	3.27	SW
				*qSHL_*5.1*_*	SHL-8DAS	3.4	6	0.072	SW
				*qSFW_*5.1*_*	SFW-8DAS	2.2	4	0.079	SW
				*qSDW_*5.1*_*	SDW-8DAS	4.5	7.9	0.00	–
				*qTDW_*5.1*_*	TDW-8DAS	2.8	5	−0.001	MO
5	id5003638	id5007323-id5013100	3–29.6	*qEV_*5.2*_*	EV1	3.7	6.6	2.5	SW
				*qEV_*5.2*_*	EV2	4.2	7.5	2.88	SW
				*qEV_*5.2*_*	EV3	3.3	6.0	1.587	SW
				*qEUE_*5.2*_*	EUE	10.7	12.6	2.531	SW
				*qSHL_*5.2*_*	SHL-8DAS	5	8.8	0.556	SW
				*qSL_*5.2*_*	SL-21DAS	4.9	8.5	−0.067	MO
				*qSFW_*5.2*_*	SFW-21DAS	3	5.4	0.002	SW
6	id6011613	ud6000218-id6007312	11.7–27.6	*qEV_*6.1*_*	EV1	6.0	10.6	1.98	SW
				*qEV_*6.1*_*	EV2	5.9	10.3	1.95	SW
				*qEV_*6.1*_*	EV3	5.6	9.7	1.648	SW
				*qEUE_*6.1*_*	EUE	7.4	12.5	2.539	SW
				*qSHL_*6.1*_*	SHL-21DAS	2.5	4.1	−1.29	MO

*EV1, Early vigor (June WS2014); EV2, Early vigor (Aug WS2014); EV3, Early vigor (June WS2015); EUE, early uniform emergence; SHL, shoot length; SL, stem length; SFW, shoot fresh weight; TFW, total fresh weight; SDW, shoot dry weight; RDW, root dry weight; TDW, total dry weight; 8DAS, 8 days after sowing; 21DAS, 21 days after sowing*.

a Chromosome on which QTL is located;

b The estimated map position in centimorgan;

c Maximum-likelihood (LOD) score for the QTL;

d The variance (%) explained by the individual QTL;

e *Positive “+” value indicates that the allele from Swarna and Negative“−”value indicates that the allele from Moroberekan, increases phenotypic values*.

QTL analysis of remaining early vigor related traits was carried out at 8 and 21 DAS. Four QTLs for shoot length (*qSHL*_*3.1*_, *qSHL*_*5.1*_, *qSHL*_*5.2*_, and *qSHL*_*6.1*_) were identified on chromosomes 3, 5(2), and 6, which explained phenotypic variances of 6.1, 6, 8.8, and 4.1%, respectively. The QTLs *qSHL*_*3.1*_, *qSHL*_*5.1*_, and *qSHL*_*5.2*_ were detected at 8 DAS, while *qSHL*_*3.1*_ and *qSHL*_*6.1*_ were expressed at 21 DAS. For stem length two QTLs (*qSL*_*3.1*_ and *qSL*_*5.2*_) were detected for stem length at chromosomes 3 and 5 with phenotypic variances of 7 and 8.5%, respectively. *qSL*_*3.1*_ was detected at 8 DAS and *qSL*_*5.2*_ at 21 DAS, indicating that *qSL*_*3.1*_ influences stem elongation during 8 DAS while *qSL*_*5.2*_ does the same during 21 DAS.

Two QTLs were detected for SFW (*qSFW*_*3.1*_ and *qSFW*_*5.2*_) on chromosomes 3 and 5 with maximum phenotypic variances of 6.1 and 5.4%, respectively. *qSFW*_*3.1*_ was detected at both 8 DAS and 21 DAS while *qSFW*_*5.2*_ was detected only at 8 DAS (Table 1).For SDW one QTL (*qSDW*_*5.1*_) was detected at 8 DAS on chromosome 5, which explained a phenotypic variance of 7.9%. The QTL (*qTFW*_*3.1*_) for TFW was detected at 8 DAS on chromosome 3, which explained phenotypic variance of 5.7%. The QTL (*qRDW*_*3.1*_) for RDW was identified at 21 DAS on chromosome 3, which explained 8.4% of the phenotypic variation (Table 1). One QTL was detected for TDW (*qDTY*_*5.1*_) on chromosome 5 with phenotypic variance of 5.

#### “QTL hotspots” for early vigor and related traits

QTL hotsopts are regions of the genome wherein groups of traits co-localize. The highest concentration of QTLs was identified in the marker interval of id3001701-id3008333 on chromosome 3 and is designated as “QTL hotspot A” with length of 13.4 mb. QTL hotspot A harbors QTLs for early vigor, early uniform emergence, shoot length, stem length, SFW, TFW, and RDW. Another sets of QTL rich region was found on chromosome 5 in the marker interval of wd5002636-id5001470 and is designated as “QTL hotspot B” with length of 0.7mb. It harbors QTLs for early vigor, early uniform emergence, shoot length, SFW, SDW, and TDW (Figures 1, 2, Table 1).

### Identification of putative candidate genes and mutations

Only QTLs which were repeatedly identified in multiple environments were used for gene identification. QTL region flanking SNP markers id3010740-id3008333 and id3010173-id3013447 on chr3, id4012189-id4004461 on chr4, wd5002636-id5001470 and id5007323-id5013100 on chr5, and ud6000218-id6007312 on chr6 were selected for this analysis. A total of 10 genes were found in the QTL regions identified on chromosome 3, 4, and 5 (Table 2). Three genes, namely glutamate decarboxylase, ethylene-insensitive 3 (*EIN3*), and expansin precursor were identified on chromosome 3. Four genes, namely alpha (α)-amylase precursor, glutamate decarboxylase, and two of phenylalanine ammonia-lyase were identified in the QTL region on chromosome 4. Three genes—phenylalanine ammonia-lyase and two of expansin precursor—were identified in the QTL region on chromosome 5 (Table 2). SNP analysis of all 10 genes among both the parents was done and three mutations are detected in the alpha-amylase precursor and ethylene-insensitive 3 gene.

**Table 2:** **Possible candidate genes identified from the QTL regions associated with early vigor and related traits**.

**S.No**.	**Chr**.	**Gene**	**Locus**	**Base pair**	**Start**	**End**
1	3	Glutamate decarboxylase	Os03g0236200	2,957	7,230,405	7,233,361
2	3	Ethylene-insensitive 3	Os03g0324300	9,433	11,820,185	11,829,617
3	3	Expansin precursor	Os03g0428700	1,012	18,570,738	18,571,749
4	4	alpha-amylase precursor	Os04g0403300	5,370	20,025,314	20,030,683
5	4	Glutamate decarboxylase	Os04g0447800	2,163	22,728,745	22,730,907
6	4	Phenylalanine ammonia-lyase	Os04g0518100	3,182	26,305,431	26,308,612
7	4	Phenylalanine ammonia-lyase	Os04g0518400	2,640	26,325,630	26,328,269
8	5	Expansin precursor	Os05g0276500	1,781	11,404,176	11,405,956
9	5	Phenylalanine ammonia-lyase	Os05g0427400	2,151	20,960,453	20,962,603
10	5	Expansin precursor	Os05g0477600	1,919	23,542,813	23,544,731

### Trait relationships and identification of best genotypes

**Figure 3:**
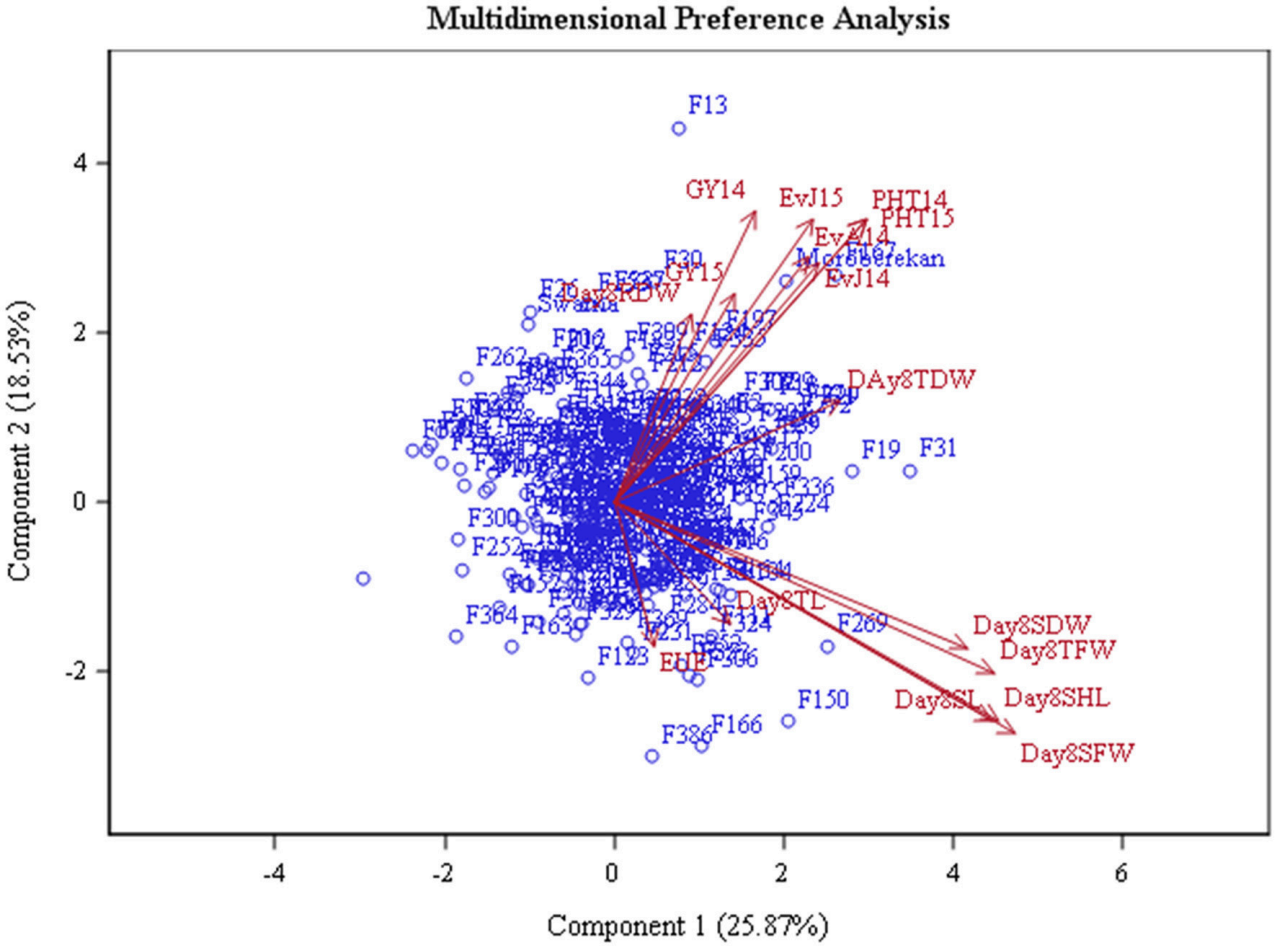
**Multi-dimensional preference biplot of phenotypic data at 8 DAS**.

**Figure 4:**
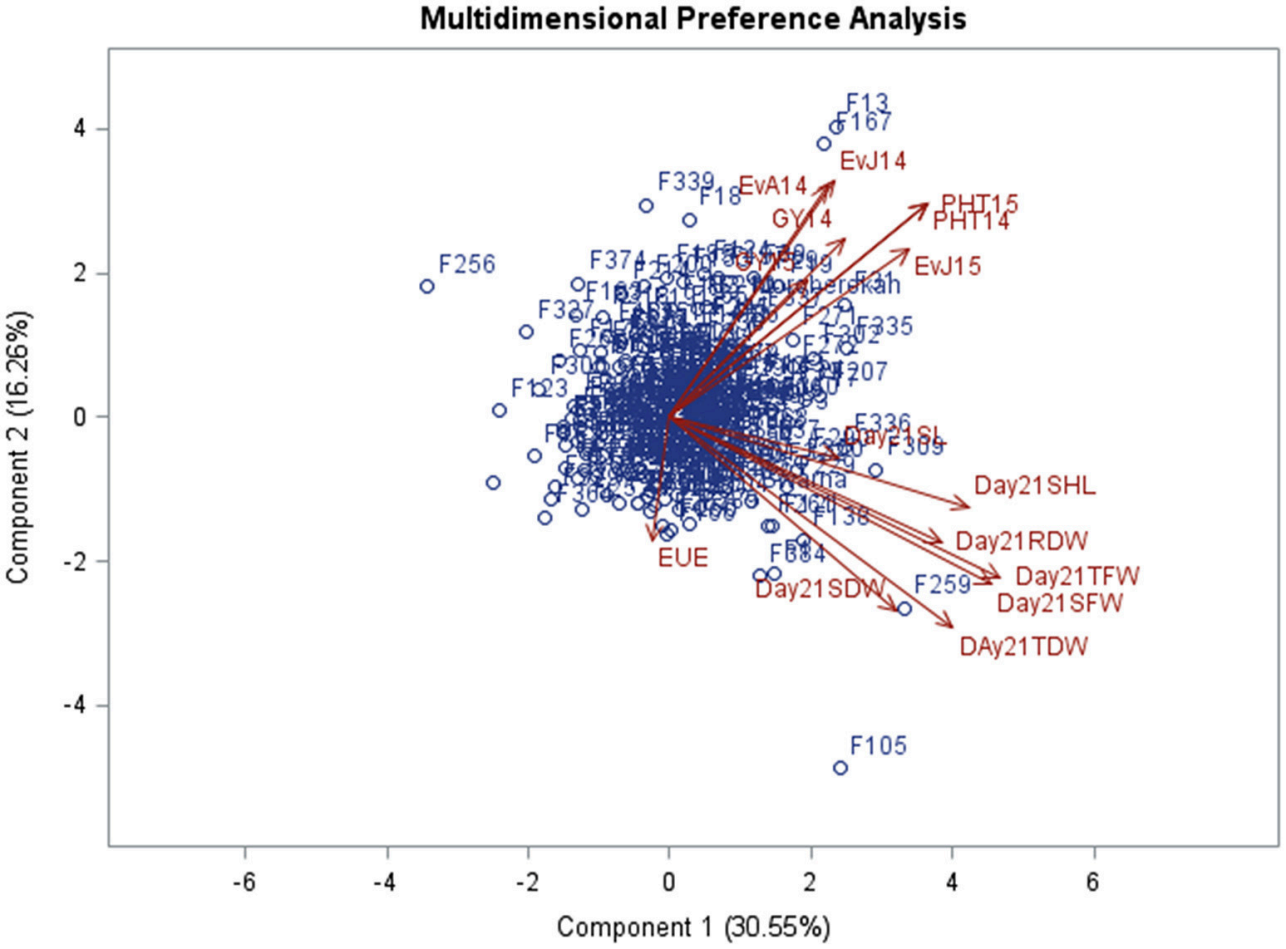
**Multi-dimensional preference biplot of phenotypic data at 21 DAS**.

MDPREF was used to study relationships among genotypes and traits. Separate biplots were drawn for the data recorded in glasshouse at 8 DAS and 21 DAS in order to investigate the consistency of trait behavior over the growing period (Figures 3, 4, Table 3). The analysis yielded two clearly interpretable dimensions that underlie the data—the first on the physiological traits (SHL, SL, TFW, SFW, SDW, TFW, TDW, and RDW) from glasshouse and the second on agronomic traits (EVA14, EVJ14, EVJ15, PHT14, PHT15, GY14, and GY15) from field. Most of the variability is covered by the first two preference axes. On the biplot, the trait scores are joined to the origin by the trait vectors. The vectors corresponding to early vigor, grain yield kilograms per hectare (GYKGPHA), and plant height (PHT) were positioned closely on the top right space while all vectors corresponding to the physiological traits as well as early uniform emergence tended toward the bottom right space. The positioning of the trait vectors was similar both at 8 and 21 DAS except that RDW and TDW were clustered along with vigor and yield vectors on the 8 DAS biplot.

**Table 3:** **Means of the best performers for (i) EV, EUE, and grain yield and (ii) physiological traits measured at 8 and 21 DAS**.

**Promising Lines**	**Shoot length**		**Stem length**		**Total fresh weight**		**Shoot fresh weight**		**Total dry weight**		**Shoot dry weight**		**Plant Height**		**Grain yield (kg/ha)**		**Early vigor**			**Early Uni. Emer**
	**8DAS**	**21DAS**	**8DAS**	**21DAS**	**8DAS**	**21DAS**	**8DAS**	**21DAS**	**8DAS**	**21DAS**	**8DAS**	**21DAS**	**WS14**	**WS 15**	**WS 14**	**WS 15**	**Jun14**	**Aug14**	**Jun15**	
**(A) EV,EUE, Yield**																				
IR91648-B-13-B (F13)	7.70	15.65	3.12	5.37	0.049	0.096	0.023	0.063	0.010	0.019	0.004	0.013	99	111	4,841	7,225	9	7	7	4
IR91648-B-124-B (F124)	7.60	14.40	3.35	4.75	0.056	0.077	0.028	0.053	0.011	0.014	0.005	0.011	87	83	5,182	7,183	5	6	7	3
IR91648-B-153-B (F153)	5.60	12.90	3.22	3.85	0.039	0.073	0.019	0.047	0.010	0.015	0.004	0.009	83	84	4,558	4,492	7	6	7	5
IR91648-B-335-B (F335)	7.65	19.25	4.10	5.36	0.052	0.11	0.027	0.072	0.008	0.020	0.004	0.014	105	106	4,550	5,183	5	7	7	5
IR91648-B-167-B (F167)	8.65	14.9	4.05	4.50	0.060	0.097	0.035	0.062	0.009	0.018	0.005	0.013	105	100	4,172	7,429	8	8	7	3
IR91648-B-183-B (F183)	6.20	12.4	2.95	3.53	0.05	0.076	0.025	0.049	0.011	0.016	0.004	0.010	78	78	4,578	4,792	7	7	5	6
IR91648-B-197-B (F197)	8.08	12.55	3.65	3.78	0.047	0.083	0.026	0.055	0.012	0.017	0.006	0.012	88	83	5,652	4,775	7	6	7	5
IR91648-B-302-B (F302)	7.92	18.03	4.12	5.05	0.056	0.107	0.035	0.068	0.008	0.020	0.005	0.014	105	76	4,505	5,017	6	7	5	6
**(B) 8 DAS**																				
IR91648-B-306-B (F306)	10.13	11.65	4.33	3.63	0.064	0.066	0.038	0.045	0.008	0.015	0.006	0.010	67	64	4,043	2,500	3	5	3	6
IR91648-B-150-B (F150)	10.53	14.43	4.05	3.73	0.076	0.090	0.043	0.061	0.011	0.018	0.009	0.013	73	91	2,252	2,500	3	5	3	6
IR91648-B-269-B (F269)	11.48	13.43	4.42	3.65	0.089	0.089	0.047	0.059	0.009	0.017	0.006	0.012	69	59	3,451	4,317	4	5	5	5
IR91648-B-324-B (F324)	7.95	13.63	4.20	3.68	0.072	0.096	0.039	0.064	0.012	0.019	0.007	0.013	65	72	2,331	2,275	6	5	3	6
IR91648-B-104-B (F104)	9.76	13.36	4.56	3.75	0.067	0.106	0.036	0.069	0.013	0.020	0.006	0.014	79	71	5,611	5,450	2	2	3	3
**(C) 21 DAS**																				
IR91648-B-138-B (F138)	10.58	18.05	5.275	5.58	0.064	0.128	0.037	0.077	0.009	0.023	0.005	0.016	88	86	3,033	3,683	3	3	5	5
IR91648-B-259-B (F259)	7.05	20.48	3.23	5.23	0.046	0.145	0.022	0.082	0.011	0.027	0.006	0.016	71	98	4,025	3,838	3	4	7	2
IR91648-B-309-B (F309)	6.73	21.25	2.98	5.60	0.052	0.123	0.025	0.087	0.009	0.025	0.004	0.019	104	82	4,694	4,517	5	5	5	4
IR91648-B-121-B (F121)	8.53	17.10	4.16	5.05	0.061	0.127	0.035	0.067	0.011	0.023	0.006	0.016	76	67	3,795	1,375	5	5	5	3

*(A) Lines selected based on early vigor, early uniform emergence and yield; (B) lines selected based on physiological traits at 8 DAS; (C) lines selected based on physiological traits at 21 DAS. EV, early vigor; EUE, early uniform emergence; DAS, days after sowing; PHT, plant height; GYKGPHA, grain yield kilograms per hectare; Names within parenthesis are abbreviations of the genotypes*.

## Discussion

### Co-localization of QTLs

Five of the six QTLs, identified for EV namely *qEV*_*3.2*_, *qEV*_*4.1*_, *qEV*_*5.1*_*, qEV*_*5.2*_, and *qEV*_*6.1*_ were constantly detected across all the three field experiments, showing the consistency of the newly-identified QTLs in this study (Figures 1, 2). The QTLs *qEV*_*3.1*_, *qEV*_*3.2*_, and *qEV*_*5.1*_ coincided well with QTLs identified by earlier researchers (Zhang et al., 2005a; Lu et al., 2007; Zhou et al., 2007; Xie et al., 2014). *qDTY*_*3.1*_, reported for grain yield under drought (Dixit et al., 2014), overlapped with *qEV*_*3.2*_. Overlapping of these QTL regions might be one of the reasons for making this genotype suitable for less frequent irrigation which is the main component of DSR system. However, recent studies reported the existence of tradeoff between vigor and drought resistance (Rebolledo et al., 2012, 2013). The co-localization of early vigor with drought QTL may makes this phenomenon less possible.

Many QTLs for traits such as seed dormancy (Miura et al., 2002), germination rate, shoot/RDW, and for physiological traits like reducing sugar and amylase activity (Cui et al., 2002; Zhang et al., 2005a) were mapped in a region similar to *qEUE*_*5.1*_. This indicates that these physiological components might have a direct relationship with seed vigor and may possibly supply energy for fast and uniform seedling growth. QTL analysis showed that the positive allele for this trait is contributed by Swarna (Table 1).

The expression of *qSHL*_*3.1*_, the QTL for shoot length during both growth stages indicates that this QTL influences shoot elongation throughout the vegetative growth stage while the other QTLs are stage specific. QTLs for total length were also reported on chromosomes 3 and 5 in a double haploid population developed from CT9993/IR62266 (Kanbar et al., 2006). The QTLs, *qSHL*_*3.1*_ and *qSHL*_*5.1*_ identified in the present study overlapped with earlier identified QTLs for total length (*qTL3-1* and *qTL5-1*; Kanbar et al., 2006).

*qSL*_*3.1*_ was detected at 8 DAS for stem length and *qSL*_*5.2*_ at 21 DAS, indicating that *qSL*_*3.1*_ influences stem elongation during 8 DAS while *qSL*_*5.2*_ does the same during 21 DAS. The marker interval RM87-RM334 was reported in roughly the same genomic region shared by presently identified QTL *qSL*_*5.2*_ (Diwan et al., 2013). Similarly, in an association mapping analysis, RM5475 and RM480 markers reported at chromosomes 3 and 5 (Dang et al., 2014) were found to overlap with these two newly-identified QTLs. Kanbar et al. (2006) also indicated two QTLs, *qphl-3-1* and *qphl-3-2*, in the same genomic region of *qSL*_*3.1*_ as found in the present study. Zhang et al. (2005a) reported that the QTL *qSV-3-1* for shoot length shared the same genomic region with *qSL*_*3.1*_.

*qSFW*_*3.1*_ for SFW was detected at both 8 DAS and 21 DAS while, *qSFW*_*5.2*_ was detected only at 8 DAS, indicating that it influences SFW only during the initial growth stage of the seedlings. *qTFW*_*3.1*_ and *qTFW*_*5.1*_ were detected for TFW at 8 DAS on chromosomes 3 and 5 (Table 1). Only one QTL (*qSDW*_*5.1*_) was detected for SDW at 8 DAS on chromosome 5. This QTL did not express at 21 DAS, implying that it influences SDW only during the initial stages of seedling growth. Single QTL (*qRDW*_*3.1*_) for RDW was identified at 21 DAS on chromosome 3. For TDW one QTL (*qTDW*_*5.1*_) was identified on chromosome 5. The QTL for TDW (*qTDW*_*5.1*_) mapped in present study were also reported by Cui et al. (2002) and Diwan et al. (2013) indicating conservation of genomic region across species.

QTLs for early vigor related traits such as SHL, SL, SFW, SDW, TFW, RDW, and TDW were stage specific (expressed either at 8 or 21 DAS), influencing plant growth at specific stages. In a similar finding, out of 21 and 16 QTLs identified for early vigor trait at 6 and 12 DAS, respectively, only three QTLs expressed for this trait at both stages (Cairns et al., 2009). Current finding is also in agreement with previous reports in rice as well as other crops that suggest that gene action was different at various developmental stages (Yan et al., 1998; Bian et al., 2015). Favorable alleles for early uniform emergence were contributed mostly by Swarna and favorable alleles for component physiological traits were contributed by Moroberekan (Table 1). This implies that both parents could possess positive genes for the traits at different loci and more superior genotypes could be developed from a recombination of positive genes contributed by both parents.

### Comparisons with previously mapped QTLs

We compared the currently identified QTL regions with previously reported loci by using whole-genome marker resources for rice based on the Gramene website (http://gramene.org). Thus, out of 23 QTLs identified in present study, the positions of 9 QTLs; three for early vigor (*qEV*_*31*_, *qEV*_*3*__*.2*_, *qEV*_*5*__.*1*_), one for uniform emergence (*qEUE*_*3*__.*1*_), two for stem length (*qSL*_*3*__.*1*_, *qSL*_*5*__.2_), and two for shoot length (*qSHL*_*3*__.*1*_, *qSHL*_*5*__.*1*_), and one for TDW (*qTDW*_*5.1*_) either overlapped or were co-localized with those reported in previous studies. Except for the 9 QTLs mentioned above, the remaining 15 (3 for EV, 5 for EUE, 1 for SL, 2 for SFW, 2 for TFW, and 2 for RDW) were novel marker loci. These marker loci were found co-localized with each other (Figures 1, 2). Co-localization of QTL for early vigor and related traits could give additional evidence on the role of the corresponding genes in seedling growth. More interestingly, the alleles for the studied traits were contributed by both parents. This indicates that the extensive level of genetic diversity for early vigor and related traits that exists among rice genotypes can be successfully exploited to improve both the traits. Overlapping of QTLs indicates the existence of a partly common genetic base which might be the pleotropic effects of a single and/or limited number of QTLs or tightly linked loci controlling these traits (Davar et al., 2011).

### “QTL hotspots” for early vigor and related traits

Genomic region associated with many traits are biologically very interesting since they may harbor influential regulators. It indicates the location of a single gene with pleiotropic effect or tightly linked loci affecting two or more traits (Hittalmani et al., 2002; Pelgas et al., 2011). In the present study the shared genomic region is referred as hotspot A and hotspot B. These were not only detected in the field experiment but also showed effect on the traits measured in the glasshouse. This shows the stability of these QTLs. Roughly the same genomic region on chromosomes 3 and 5 was shared by QTLs identified in different mapping populations of rice (Zhang et al., 2005a; Lu et al., 2007; Zhou et al., 2007). Such QTLs need to be further characterized and used as potential target in marker-assisted breeding for improving rice varieties for early vigor trait. Cui et al. (2002) identified several seedling vigor QTLs under controlled laboratory and glasshouse conditions, with most of them sharing a common genomic region. Thus, it is suggested that QTLs hotspots identified in multiple environment could be ideal for understanding its regulatory role and further in molecular breeding.

### Putative candidate genes and mutations

For localizing trait related genes, QTL analysis is a powerful method but identifying the causal gene remains difficult because of instability in the expression of QTLs across multiple environment. Stable QTLs identified in present study were used for candidate gene identification. A total of 10 genes were found in the QTL regions identified on chromosome 3, 4, and 5 (Table 2). Glutamate decarboxylase identified on chromosome 3 and 4 has role in the growth of young tissues of seedlings (Oh and Choi, 2001). Ethylene insensitive 3 (*EIN3*) identified on chromosome 3 is a master regulator of ethylene signaling pathway (Yang et al., 2015). Ethylene plays a very important role in the growth and development of the plant. Ethylene is also involved in cell differentiation processes including differential cell elongation (Cervantes, 2006) and its effect on cell elongation is the probable reason for its role in fast seedling germination and growth. Expansin identified on chromosome 3 and 5 has a role in coleoptile elongation under anaerobic conditions for rice. High levels of the expansin gene were earlier reported in leaves, mesocotyls, and coleorhizae of young seedlings, demonstrating its indispensible role in the growth of rice tissues (Huang et al., 2000).

The role of phenylalanine ammonia-lyase (identified on chromosome 4) in seedling growth has been reported as well (de Cássia Siqueira-Soares et al., 2013). Alpha-amylase in the aleurone layer hydrolyses the endosperm starch into sugar which provides energy for the proper growth of roots and shoots (Beck and Ziegler, 1989). Rice cultivars with α-amylase activity showed a higher germination rate and faster seedling growth at the early stage (Krishnasamy and Seshu, 1989). The role of these genes is discussed earlier in this section. The presence of the potential genes (phenylalanine ammonia-lyase, expansin precursor) in the identified QTL regions might account for the early growth of seedlings. Moreover, validation of these genes will give a clearer insight about their role in facilitating fast emergence and efficient early seedling growth.

Mutations are main cause of change in phenotype of organisms. In present study all 10 genes identified were subjected for SNP analysis among both the parents. One mutation was identified in alpha-amylase precursor (LOC_Os04g33040) at base pair position 20006622 bp (T/G) and two mutation in Ethylene-insensitive 3 (LOC_Os03g20790) gene at basepair position of 11776924 (T/C) and 11777241 (G/N). This mutation can lead to change in the structure of encoded protein or to complete loss or decrease in its expression.

### Trait relationships and identification of best genotypes

A number of studies have used Principle Component Analysis (PCA) and its biplots in investigating phenotypic correlations between traits in rice and in other crops (Oladejo et al., 2011; Tabrizi et al., 2011; Mishra et al., 2015). However, Multidimensional Preference Analysis (MDPREF) has an advantage over PCA in that non-quantitative traits like early vigor can be accommodated and, at the same time, relationships among genotypes and traits can be presented on the same space (Seligson, 1977; Fernandez et al., 2005). Each trait vector on the biplot is approximately in the direction of the genotypes that are most “preferred” for the trait and away from the genotypes that are least “preferred.” When the genotype points are projected onto the trait vectors, the order of projected points on the vectors will correspond optimally to the genotype's rank for the trait. For instance, genotypes F306 (IR 91648-B-306-B), F150 (IR 91648-B-150-B), F269 (IR 91648-B-269-B), F324 (IR 91648-B-324-B), and F104 (IR 91648-B-104-B) were preferred in terms of physiological traits at 8 DAS (Figure 3, Table 3). However, these genotypes were not the best at 21 DAS as they showed poor to moderate vigor and below average yield. Genotypes F138 (IR 91648-B-138-B), F259 (IR 91648-B-259-B), F309 (IR 91648-B-309-B), and F121 (IR 91648-B-121-B) were preferred for physiological traits at 21 DAS (Figure 4). These entries showed moderate to good early vigor and moderate to good yield. Genotypes F13 (IR 91648-B-13-B), F124 (IR 91648-B-124-B), F153 (IR 91648-B-153-B), F335 (IR 91648-B-335-B), F167 (IR 91648-B-167-B), F197 (IR 91648-B-197-B), and F302 (IR 91648-B-302-B) were in the direction of the vectors corresponding to yield and early vigor trait as they were good yielding and possessed good early vigor (Figures 3, 4). Thus, lines that possessed moderately good physiological traits showed good early vigor and yield. These results were in line with previous reports (Cui et al., 2002; Kumar et al., 2009; Namuco et al., 2009) which indicated that plants with good early vigor tended to have good yield.

### Implication of selected lines for breeding rice for higher yield under dry DSR

QTL hotspots A and B identified in the present study harbor almost all traits studied here. These QTL hotspot regions can be effectively used in routine MAS breeding programs. Reports have suggested that early vigor traits and high yield can be combined, meaning that these complex traits do not present a negative (genetic or physiological) linkage (Zhao et al., 2006; Namuco et al., 2009). Lines with strong early vigor and fast uniform emergence across all three environments, coupled with good yield, were selected (Table 3) with the objective to genetically improve rice genotypes for early vigor. This is to achieve uniform plant population in the field through efficient and quick seedling germination from soil depths and early vigor to aid weed reduction under dry DSR conditions. The selected lines (especially IR 91648-B-167-B and IR 91648-B-13-B) which have strong early vigor, fast uniform emergence, and good yield can be used as donors in the development of new rice varieties with higher yield under DSR.

## Conclusion

This study identified six genomic regions for early vigor and related traits, among them two were QTL hotspots (QTL hotspot A and QTL hotspot B) which harbor almost all target traits. QTL hotspot A contain QTLs for early vigor, early uniform emergence, shoot length, stem length, SFW, RDW, TFW, and QTL hotspot B harbor QTLs for early vigor, early uniform emergence, shoot length, SFW, and SDW. Co-localization of QTLs for different traits suggests a common genetic basis of regulation of the traits and point to the regulation of these traits either by tightly linked genes or by pleotropic regulation. The expression of a QTL for the measured traits was not necessarily the same at 8 and 21 DAS, indicating that the genes regulating seedling growth at different stages may be different. Comparisons with published studies revealed that most of these regions were previously identified in different genetic backgrounds and could potentially be used as introgression targets in MAS. The list of plausible candidate genes and mutations from the present study will facilitate further verification and experimental evaluation. An assessment of the phenotypic relationships between early vigor and related traits indicated strong correlations between early vigor and grain yield and moderate associations between early uniform emergence and the physiological traits. The analysis also identified lines from the Swarna^*^3/Moroberekan population that were best for early vigor and agro-physiological traits and which be used in marker-assisted breeding programs.

## Author contributions

US Conducted experiment and wrote the manuscript. SY, SD, PR, MD helped in experimental work, and contributed to the manuscript modification. KR conducted the statistical analysis and wrote the statistical conclusion of the manuscript finding and contributed to manuscript revision. AK conceived and designed the work and contributed to the manuscript revision. All authors read and approved the final manuscript.

### Conflict of interest statement

The authors declare that the research was conducted in the absence of any commercial or financial relationships that could be construed as a potential conflict of interest.

## Abbreviations

QTLs: Quantitative trait loci
DSR: Direct-seeded rice
TPR: Transplanted-puddled rice
WS: Wet seasons
SSD: Single seed descent
DAS: Day after sowing
SAS: Standard Evaluation System
CIM: Composite interval mapping
LOD: Logarithm of odds
MDPREF: Multi-dimensional preference analysis
PCA: Principal component analysis.
