# Supplementary material for: QTL Hotspots for Early Vigor and Related Traits under Dry Direct-Seeded System in Rice (Oryza sativa L.)
Source: Front Plant Sci. 2017 Mar 2;8:286. doi: 10.3389/fpls.2017.00286 (PMC5332406; doi:10.3389/fpls.2017.00286)
Supplement: Supplementary file 1 [file Table1.DOCX]

**Supplementary Table 1. Experimental site description.**

| **Year** | 2014 | 2015 |
| --- | --- | --- |
| **Season** | Wet | Wet |
| **pH** | 7.96 | 7.68 |
| **EC (ds/m)** | 0.18 | 0.11 |
| **OC (%)** | 0.69 | 0.66 |
| **Avail-P (ppm)** | 9.69 | 7.48 |
| **Exch-K (ppm)** | 193 | 132 |
| **Exch-Na (ppm)** | 140 | 71 |
| **Avail-Fe (ppm)** | 11.1 | 14.94 |
| **Avail-Zn (ppm)** | 2.2 | 2.84 |
| **Rainfall (mm)** | 494.1 | 727.7 |
| **Ave. Max temperature (**°C) | 32.25 | 32.67 |
| **Ave. Min temperature (**°C) | 19.73 | 19.71 |
| **Relative humidity (%)** | 64.06 | 66.76 |

EC-electrical conductivity; OC- Organic carbon; ppm- parts per million.
